# Supplementary material for: Clinical and pharmacological factors associated with mortality in patients with COVID-19 in a high complexity hospital in Manaus: A retrospective study
Source: PLoS One. 2023 Feb 10;18(2):e0280891. doi: 10.1371/journal.pone.0280891 (PMC9916623; doi:10.1371/journal.pone.0280891)
Supplement: S1 Table — (DOCX) [file pone.0280891.s001.docx]

**S1 Table. Multiple logistic regression analysis of risk factors for mortality in hospitalized patients with COVID-19.**

| **Variables** | **OR** | **SE** | **p** | **Confidence Interval (95%)** | |
| --- | --- | --- | --- | --- | --- |
|  |  |  |  | Lower | Upper |
| Age | 1.046 | 0.015 | 0.002 | 1.017 | 1.076 |
| Number of comorbidities | 1.542 | 0.279 | 0.017 | 1.082 | 2.200 |
| Length of stay | 0.934 | 0.015 | < 0.001 | 0.905 | 0.964 |
| ICU admission | 3.370 | 2.324 | 0.078 | 0.872 | 13.020 |
| VM | 19.298 | 13.297 | < 0.001 | 5.000 | 74.476 |
| Corticosteroids | 0.943 | 0.732 | 0.940 | 0.206 | 4.318 |
| antimicrobials | 0.590 | 0.444 | 0.484 | 0.135 | 2.582 |
| Antifungals | 2.206 | 1.364 | 0.201 | 0.656 | 7.414 |
| Penicillin | 3.025 | 1.477 | 0.023 | 1.161 | 7.877 |
| Glycopeptides | 2.761 | 1.501 | 0.062 | 0.952 | 8.012 |
| Anticoagulants | 2.099 | 2.374 | 0.512 | 0.229 | 19.268 |
| Oxygen Saturation | 0.955 | 0.042 | 0.302 | 0.875 | 1.042 |
| AST | 0.999 | 0.002 | 0.803 | 0.996 | 1.003 |

SE: standard error MV: mechanical ventilation ICU: Intensive Care Unit AST: Aspartate Aminotransferase
