# Supplementary material for: Clinical and pharmacological factors associated with mortality in patients with COVID-19 in a high complexity hospital in Manaus: A retrospective study
Source: PLoS One. 2023 Feb 10;18(2):e0280891. doi: 10.1371/journal.pone.0280891 (PMC9916623; doi:10.1371/journal.pone.0280891)
Supplement: Attachment — Submitted filename: review.docx [file pone.0280891.s002.docx]

Badin R. et al reported a retrospective study of clinical and pharmacological factors with mortality in patients with COVID-19 in a high complexity hospital in Manaus, Brazil. They included patients from a reference hospital belonging to the Brazilian public health system, in Manaus, from March 2020 to July 2021 and analyzed the clinical and demographic features, the presence of comorbidities, and pharmacotherapeutic management in patients hospitalized for diagnosis of COVID-19, in addition to identifying predictive factors for mortality. After multivariate logistic regression analysis, age, need for mechanical ventilation, length of hospital stay, and penicillin use were associated with death. The topic is interesting and has important role in clinical application. However, some issues are still needed to addressed:

1. As we all know, different genotypes of SARS-CoV-2 has different ability of infection and pathogenesis. The authors should also analyze the genotypes influence on the mortality.
2. It is important to analyze the laboratory parameters (such as CRP, ALT, AST, pCO2, pO2, etc.), and radiological findings at admission with relationship with mortality.
3. It is also important to analyze the demographic and laboratory parameters to find multivariate predictors of in-hospital mortality of patients with COVID-19.
4. Please check and modify the format of the references one by one.
