# Supplementary material for: Clinical and pharmacological factors associated with mortality in patients with COVID-19 in a high complexity hospital in Manaus: A retrospective study
Source: PLoS One. 2023 Feb 10;18(2):e0280891. doi: 10.1371/journal.pone.0280891 (PMC9916623; doi:10.1371/journal.pone.0280891)
Supplement: Attachment — Submitted filename: responsestoreviewer.docx [file pone.0280891.s003.docx]

January 07, 2023

To

C. Emily Chenete, PhD,

Editor-in-Chief of Plos one,

Dear Dr. Chenete and reviewers,

We are pleased to forward the responses regarding the observations by the reviewers of the manuscript **“Clinical and pharmacological factors associated with mortality in patients with COVID-19 in a high complexity hospital in Manaus: a retrospective study”,** co-authored by Rebeka Caribé Badin, Robson Luís Oliveira de Amorim, Alian Aguila, Liliane R. A. Manaças, which we submit for publication in *Plos one***.**

1. As we all know, different genotypes of SARS-CoV-2 has different ability of infection and pathogenesis. The authors should also analyze the genotypes influence on the mortality.

During the study period, the genotypic characterization of the SARS-COV-2 strains was carried out by sampling, making it impossible to analyze the impact of this variable on the mortality rate. However, data from the literature (ref: 23-25) and from the epidemiological bulletin of the city of Manaus (ref: 7) report the predominance of the B.1.195 variant in the first wave of infection (June and November 2020) and the P.1 variant in the second wave (December 2020 and February 2021). This information is described in the article discussion.

2. It is important to analyze the laboratory parameters (such as CRP, ALT, AST, pCO2, pO2, etc.), and radiological findings at admission with relationship with mortality.

Recognizing that laboratory and image parameters are also extremely relevant to guide the clinical decision and considering the reviewer's suggestion, we included in the study the analysis of the AST, ALT, and oxygen saturation variables. However, due to the lack of data in the patient’s medical records, it was not possible to include the other suggested variables in the study. The analysis of the collected parameters was incorporated into the reviewed version of the manuscript.

3. It is also important to analyze the demographic and laboratory parameters to find multivariate predictors of in-hospital mortality of patients with COVID-19.

All of parameters included in the research (demographic, clinical, laboratory, and pharmacological) were statistically analyzed. For quantitative variables, the Kolmogorov-Smirnov Normality test was applied. Student's t-test (Normal Distribution) and Mann-Whitney (Non-Normal Distribution) were used to compare two groups (survivors and non-survivors). Categorical variables were shown in percentages or absolute values , and the Chi-Square Test and Fisher's Exact Test were used to verify the association between categorical variables. The statistically significant variables identified in the univariate analysis were included in the multivariate logistic regression analysis (model 1) (S1).

However, some variables did not prove to be statistically significant in model one, such as the need for ICU, the use of corticosteroids, antimicrobials, anticoagulants, antifungals, and an antibiotic class of glycopeptides.

Thus, a second multivariate logistic regression analysis model was done using the reverse stepwise approach until all variables were significant (model 2) (table 3). In the article, we rewrote this information in the topic “characteristics associated with mortality” to clarify the multivariate logistic regression analysis.

4. Please check and modify the format of the references one by one.

We checked and corrected the references, according to the journal's instructions. For this purpose, we use the Zotero program.
